# Comparison of in-hospital glycemic variability and admission blood glucose in predicting short-term outcomes in non-diabetes patients with ST elevation myocardial infarction underwent percutaneous coronary intervention

**DOI:** 10.1186/s13098-017-0217-1

**Published:** 2017-03-21

**Authors:** Shu-hua Mi, Gong Su, Hong-xia Yang, Yun Zhou, Lei Tian, Tao Zhang, Hong Tao

**Affiliations:** 1 Center of Cardiology, Beijing An Zhen Hospital, Capital Medical University, No. 2 Anzhen Road, Chaoyang District, Beijing, China; 2 Department of Endocrinology, Beijing An Zhen Hospital, Capital Medical University, Beijing, China

**Keywords:** Glycemic variability, Admission blood glucose, Acute myocardial infarction, Major adverse cardiac events

## Abstract

**Aims:**

Admission hyperglycemia is associated with increased mortality and major adverse cardiac events (MACE) in patients with or without diabetes mellitus after acute myocardial infarction (AMI). However, effects of glycemic variability (GV) on outcomes of non-diabetes patients with AMI still remains unclear. The aim of this study is to compare the prognostic value of in-hospital GV with admission blood glucose (ABG) for 3-month MACE in non-diabetes patients with ST elevation myocardial infarction (STEMI) who underwent percutaneous coronary intervention (PCI).

**Methods:**

We analyzed 256 non-diabetes patients with STEMI in study. The GV accessed by mean amplitude of glycemic excursions (MAGE) was calculated from blood glucose profiles of continuous glucose monitoring system (CGMS) during hospitalization. ABG was measured on admission. Main endpoints were 3-month MACE; secondary endpoints were GRACE scores and enzymatic infarct size. Predictive effects of MAGE and ABG on the MACE in patients were analyzed.

**Results:**

In all participants, MAGE level was associated with ABG level (r = 0.242, p < 0.001). Both elevated MAGE levels (p = 0.001) and elevated ABG (p = 0.046) were associated with incidences of short-term MACE. Patients with a higher MAGE level had a significantly higher cardiac mortality (5.8 vs. 0.6%, p = 0.017) and incidence of acute heart failure (12.8 vs. 2.4%, p = 0.001) during 3 months follow-up. In multivariable analysis, high MAGE level (HR 2.165, p = 0.023) was significantly associated with incidence of short-term MACE, but ABG (HR 1.632, p = 0.184) was not. The area under the receiver-operating characteristic curve for MAGE (0.690, p < 0.001) was superior to that for ABG (0.581, p = 0.076).

**Conclusions:**

To compare with ABG, in-hospital GV may be a more important predictor of short-term MACE and mortality in non-diabetes patients with STEMI treated with PCI.

## Background

Acute hyperglycemia on admission is common in non-diabetes patients with acute myocardial infarction (AMI), and is a risk factor for increased mortality and in-hospital adverse outcomes [1, 2]. It has shown that high admission blood glucose (ABG) levels are associated with increased mortality after AMI [3, 4]. However, recent studies have shown that glycemic variability (GV) may be of prognostic value with regard to future cardiovascular events [5, 6]. Some studies showed that glucose fluctuations could play a deleterious role through the activation of oxidative stress, one of the key pathophysiological mechanisms for the development of cardiovascular complications [7, 8]. However, whether GV has the important prognostic significance of short-term major adverse cardiac events (MACE) in non-diabetes patients is unknown. In this study, we investigated the independent prognostic value of the in-hospital GV and ABG levels in patients without known diabetes mellitus who underwent percutaneous coronary intervention (PCI) for ST elevation myocardial infarction (STEMI).

## Methods

### Study population

This was a single-center, prospective follow-up study. 265 non-diabetes patients with STEMI who underwent PCI had baseline clinical and laboratory studies, including the Global Registry of Acute Coronary Events (GRACE) risk scores [9], the mean amplitude of glycemic excursions (MAGE) and ABG levels (All patients’ ABG level is less than 16.7 mmol/L). STEMI was defined as complaints of chest pain with ECG signs compatible with AMI (ST-segment elevation >2 mm in precordial leads and >1 mm in limb leads). Thrombolysis in Myocardial Infarction (TIMI) flow was scored according to the TIMI flow grading system before and after PCI. Myocardial infarct size was measured by peak creatinine kinase (CK) level in the first 24 h after admission. Diabetes mellitus was diagnosed according to the American Diabetes Association criteria or the use of insulin or glucose-lowering medication. Hypertension was defined as systolic blood pressure ≥140 mmHg and/or diastolic blood pressure ≥90 mmHg or treatment with oral antihypertension drugs. Hyperlipidemia was diagnosed according to the modified National Cholesterol Education Program-Adult Treatment Panel III. The estimated glomerular filtration rate (eGFR) value was calculated by modification of diet in renal disease equation [10].

To enable completed follow-up and repeated visits to our outpatient clinic, only patients under the age of 80 and living within the hospital’s catchment area were eligible. The exclusion criteria were severe non-cardiac disease with expected survival of less than 3 months and unwillingness to participate. A patient could only be included once. Thus, 256 patients with complete data were included in the final analysis. Patients were categorized according to MAGE and ABG value, respectively (MAGE group1: the highest tertile, MAGE >3.26 mmol/L; MAGE group2: MAGE ≤3.26 mmol/L; ABG group1: the highest tertile, ABG >7.8 mmol/L; ABG group2: ABG ≤7.8 mmol/L). Blood samples were collected on admission in Emergency department and after overnight fasting and stored at −70 °C prior to analysis. Blood glucose, creatinine, total cholesterol, and triglyceride levels were measured by automatic biochemical analyzer (Hitachi 747; Hitachi, Tokyo, Japan).

### Continuous glucose monitoring

All patients were equipped with continuous glucose monitoring system (CGMS, Medtronic MiniMed, USA), and were monitored for 72 consecutive hours after PCI. A CGMS sensor was inserted into the subcutaneous abdominal fat tissue, calibrated according to the standard Medtronic MiniMed operating guidelines. During CGMS monitoring, patients checked their blood glucose level with a self-monitoring of blood glucose (SMBG) device (Medisafe Mini, Terumo, Japan) at least 4 times per day. Then, they entered the SMBG data and time of each meal into the CGMS. After monitoring for 72 h, the recorded data were downloaded into a personal computer for analysis of the glucose profile and glycemic excursion parameters with MiniMed Solutions software. The MAGE was calculated from the intermediate 24 h of recordings to avoid bias due to insertion and removal of the CGMS or insufficient stability of the monitoring system. Since measurable range of glucose by CGMS was mechanically limited from 2.2 to 22.2 mmol/L, the case showing the data out of this range was excluded from the study. The MAGE was calculated by measuring the arithmetic mean of the differences between consecutive peaks and nadirs, provided that the differences are greater than one standard deviation of the mean glucose value [11]. Patients would avoid glucose infusion during CGMS monitoring period. Otherwise, the patient would be excluded from the study.

### Coronary intervention

All patients were performed with subsequent PCI when indicated as part of the routine treatment for all STEMI patients in Beijing An Zhen Hospital. Coronary intervention was performed using standard techniques, including percutaneous transluminal coronary angioplasty, thrombus aspiration, intracoronary stenting, and/or mechanical rotational atherectomy. The PCI strategy was at the operator’s discretion. All patients were pretreated with aspirin, heparin, and clopidogrel before PCI. After intracoronary stent implantation, all patients received aspirin and clopidogrel for at least 6 months. Other adjunctive pharmacotherapy was administered at the discretion of the operator. Repeat cardiac catheterization was performed for recurrent symptoms or objective evidence of ischemia during provocative testing. Routine angiographic follow-up was not undertaken.

### Follow-up

All patients meeting criteria for this analysis were invited to participate in the study. After informed consent was obtained from the patient or a family member, clinical follow-up was performed by telephone interview and review of hospital records. Patients were followed up prospectively for 3 months. During follow-up period, incidences of MACE were registered, including new-onset myocardial infarction, acute heart failure, repeat target vessel revascularization (TVR) after initial revascularization and cardiac death. All MACE data were adjudicated by an experienced cardiovascular physician blinded to clinical details and outcomes.

### Statistical analysis

All statistical analyses were performed by using SPSS for Windows 20.0 (SPSS Inc, Chicago, IL, USA). Data are presented as frequencies and percentages for categorical variables, median for abnormal distributed parameters and mean ± SD for continuous distributed variables, unless otherwise indicated. Differences between two groups were assessed by using the Chi square, Mann-Whitney rank analysis and unpaired *t*-tests. Correlation between continuous variables was determined by Pearson correlation coefficients. The primary end point was 3-month MACE. Secondary end points were GRACE scores and enzymatic infarct size. MAGE levels were included as continuous and as categorized (≤3.26 or >3.26 mmol/L) variables. ABG levels were also included as continuous and categorized (≤7.8 or >7.8 mmol/L) variables. The predictive values of MAGE and ABG for the presence of MACE were calculated by constructing receiver-operating characteristic (ROC) curves. To ascertain the independent contribution to MACE, multivariate regression analysis was made (Cox regression using backward stepwise variable selection methods). A value of *p* < 0.05 was considered statistically significant.

## Results

### Baseline characteristics

During the study period, 265 patients were enrolled. 256 patients (96.6%) with complete data were included in the final analysis (9 patients were removed from study, 5 for failure of CGMS monitoring and 4 for incomplete follow-up data). Mean age was 61.7 ± 6.4 years, 60.5% were male. Baseline characteristics of patient groups based on levels of MAGE and ABG are shown in Tables 1 and 2. There was a strong correlation between MAGE and ABG (Pearson r = 0.242, p < 0.001). Higher MAGE or higher ABG was older, and more often had lower values of left ventricular ejection fraction (LVEF) and eGFR. High MAGE levels were associated with more frequent presence of multivessel disease and more stents planted.

**Table 1:** Baseline characteristics in non-diabetic patients with STEMI based on MAGE levels

	MAGE (mmol/L)		p
	≤3.26	>3.26	
n	170	86	
Patient demographics			
Age (years)	61 (39–77)	64 (36–79)	<0.001
Males	99 (58.2)	56 (65.1)	0.344
BMI (kg/m^2^)	26.6 (20.3–33.1)	26.7 (22.5–41.9)	0.727
Medical history			
Prior MI	16 (9.4)	14 (16.3)	0.148
Prior PCI	26 (15.3)	14 (16.3)	0.857
Prior CABG	10 (5.9)	7 (8.1)	0.596
Risk factors			
Hypertension	112 (65.9)	61 (70.9)	0.480
Hyperlipidemia	87 (51.2)	54 (62.8)	0.085
Current smoking	82 (48.2)	47 (54.7)	0.356
LVEF (%)	55.47 ± 9.94	50.27 ± 9.89	<0.001
eGFR (mL/min/1.73 m^2^)	71.67 ± 29.05	62.55 ± 17.76	0.008
TC (mmol/L)	4.60 ± 0.95	4.67 ± 1.02	0.588
TG (mmol/L)	1.82 ± 1.39	1.90 ± 0.73	0.591
ABG (mmol/L)	7.01 ± 1.68	7.91 ± 2.20	<0.001
MAGE (mmol/L)	2.17 ± 0.73	4.28 ± 1.06	<0.001
HbA_1c_ (%)	4.71 ± 0.90	5.70 ± 1.50	<0.001
Angiographic data			
Culprit vessel, LAD	68 (40.0)	38 (44.2)	0.591
Multi-vessel CAD	78 (45.9)	53 (61.6)	0.024
TIMI grade 3 before PCI	46 (27.1)	15 (17.4)	0.120
TIMI grade 3 after PCI	163 (95.9)	78 (90.7)	0.156
Stents	1.51 ± 0.87	1.79 ± 1.09	0.027

Data are mean ± SD and number (%)

*STEMI* ST elevated myocardial infarction, *MAGE* the mean amplitude of glycemic excursions, *BMI* body mass index, *MI* myocardial infarction, *PCI* percutaneous coronary intervention, *CABG* coronary artery bypass graft, *LVEF* left ventricular ejection fraction, *eGFR* estimated glomerular filtration rate, *TC* total cholesterol, *TG* triglyceride, *ABG* admission blood glucose, *HbA*_*1c*_ glycated hemoglobin, *LAD* left anterior descending artery, *CAD* coronary artery disease

**Table 2:** Baseline characteristics in non-diabetic patients with STEMI based on ABG levels

	ABG (mmol/L)		p
	≤7.80	>7.80	
n	170	86	
Patient demographics			
Age (years)	60 (36–79)	64 (44–72)	0.011
Males	105 (61.8)	50 (58.1)	0.590
BMI (kg/m^2^)	26.6 (23.5–41.9)	26.6 (20.3–32.7)	0.233
Medical history			
Prior MI	18 (10.6)	12 (14.0)	0.420
Prior PCI	27 (15.9)	13 (15.1)	1.000
Prior CABG	11 (6.5)	6 (7.0)	1.000
Risk factors			
Hypertension	114 (67.1)	59 (68.6)	0.888
Hyperlipidemia	92 (54.1)	49 (57.0)	0.692
Current smoking	88 (51.8)	41 (47.7)	0.597
LVEF (%)	55.81 ± 10.34	51.57 ± 9.80	0.017
eGFR (mL/min/1.73 m^2^)	73.54 ± 28.54	58.85 ± 16.86	<0.001
TC (mmol/L)	4.58 ± 0.96	4.71 ± 0.95	0.287
TG (mmol/L)	1.81 ± 1.39	1.93 ± 0.75	0.376
ABG (mmol/L)	6.17 ± 1.07	9.56 ± 1.27	<0.001
MAGE (mmol/L)	2.62 ± 1.15	3.40 ± 1.45	<0.001
HbA_1c_ (%)	4.76 ± 1.28	5.81 ± 1.42	<0.001
Angiographic data			
Culprit vessel, LAD	65 (38.2)	41 (47.7)	0.179
Multi-vessel CAD	82 (48.2)	49 (57.0)	0.233
TIMI grade 3 before PCI	43 (25.3)	18 (20.9)	0.535
TIMI grade 3 after PCI	162 (95.3)	79 (91.9)	0.273
Stents	1.54 ± 0.911	1.72 ± 1.04	0.170

Data are mean ± SD and number (%)

*STEMI* ST elevated myocardial infarction, *ABG* admission blood glucose, *BMI* body mass index, *MI* myocardial infarction, *PCI* percutaneous coronary intervention, *CABG* coronary artery bypass graft, *LVEF* left ventricular ejection fraction, *eGFR* estimated glomerular filtration rate, *TC* total cholesterol, *TG* triglyceride, *MAGE* the mean amplitude of glycemic excursions, *HbA*_*1c*_ glycated hemoglobin, *LAD* left anterior descending artery, *CAD* coronary artery disease

### Outcomes of patients

**Fig. 1:**
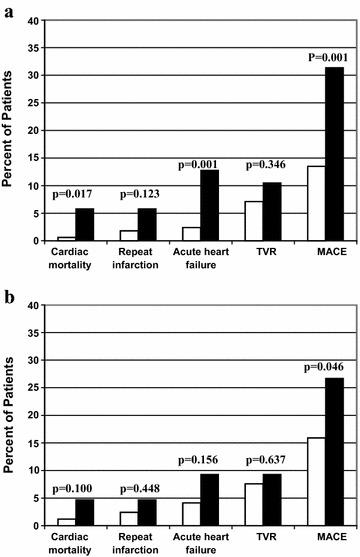
**a** Incidence of MACE after 3-month follow-up in relation to MAGE levels (*white bars* MAGE level ≤3.26 mmol/L; *black bars* MAGE level >3.26 mmol/L). **b** Incidence of MACE after 3-month follow-up in relation to ABG levels (*white bars* ABG level ≤7.80 mmol/L; *black bars* ABG level >7.80 mmol/L)

At the end of 3-month follow-up, 6 patients had died (2.3%) for cardiac causes, 8 patients had new-onset myocardial infarction (3.1%), 15 patients had acute heart failure (5.9%) and 21 patients had TVR (8.2%). Incidence of total short-term MACE was significantly higher with increasing MAGE levels and ABG levels. GRACE scores and infarct size measured by peak CK were also strongly associated with higher MAGE and ABG levels. To compare with patients of lower MAGE, higher MAGE patients had significantly higher cardiac mortality (5.8 vs. 0.6%, p = 0.017) and incidence of acute heart failure (12.8 vs. 2.4%, p = 0.001). Differences of rates of cardiac death, repeat infarction, acute heart failure and TVR were not statistically significant between two ABG groups (Table 3 and Fig. 1).

**Table 3:** Clinical outcomes of non-diabetic patients with STEMI based on MAGE and ABG

	MAGE (mmol/L)			ABG (mmol/L)		
	≤3.26	>3.26	p	≤7.80	>7.80	p
n	170	86		170	86	
Peak CK (U/L)	905 (300–4077)	1290 (471–4658)	0.020	897 (300–4428)	1372 (398–4658)	0.039
GRACE score	140 ± 33	152 ± 34	0.005	141 ± 34	150 ± 33	0.036
MACE	20 (13.5)	30 (31.4)	0.001	26 (15.9)	24 (26.7)	0.046
Cardiac death	1 (0.6)	5 (5.8)	0.017	2 (1.2)	4 (4.7)	0.100
New-onset MI	3 (1.8)	5 (5.8)	0.123	4 (2.4)	4 (4.7)	0.448
Acute HF	4 (2.4)	11 (12.8)	0.001	7 (4.1)	8 (9.3)	0.156
TVR	12 (7.1)	9 (10.5)	0.346	13 (7.6)	8 (9.3)	0.637

Data are mean ± SD and number (%)

*STEMI* ST elevated myocardial infarction, *MAGE* the mean amplitude of glycemic excursions, *ABG* admission blood glucose, *CK* creatine kinase, *MACE* major adverse cardiac events, *MI* myocardial infarction, *HF* heart failure, *TVR* repeat target vessel revascularization

### Multivariate analysis

Cox regression analysis was used to investigate the associations of MAGE and ABG with incidences of MACE with adjusting for age, sex, and all possible predictors of MACE (prior MI, hypertension, hyperlipidemia, current smoking, eGFR, LVEF, body mass index, multivessel coronary artery disease, anterior infarction, TIMI flow before PCI and TIMI flow after PCI). Results of analysis showed that MAGE (HR 2.165, 95% CI 1.114–4.219, p = 0.023), but not ABG, was significantly associated with short-term MACE. Significant predictors are presented in Table 4.

**Table 4:** Multivariate analysis of determinants of short-term MACE

Independent variables	HR	95% CI		*p* value
		Lower	Upper	
Age (per decade)	2.158	1.435	3.245	<0.001
LVEF <40%	3.137	1.543	6.380	0.002
TIMI flow after PCI < grade 3	2.984	1.276	6.980	0.012
MAGE >3.26 mmol/L	2.271	1.154	4.471	0.018

*MACE* major adverse cardiac events, *HR* hazard ratio, *CI* confidence interval, *LVEF* left ventricular ejection fraction, *TIMI* thrombolysis in myocardial infarction, *PCI* percutaneous coronary intervention, *MAGE* the mean amplitude of glycemic excursions

### ROC curve for MAGE and ABG in predicting short-term MACE

**Fig. 2:**
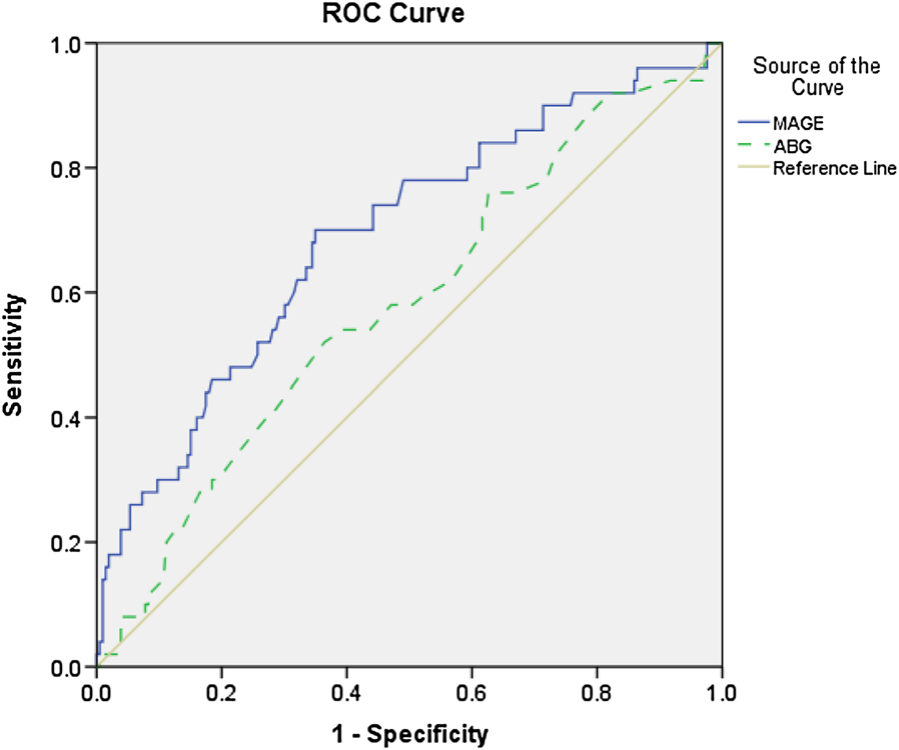
Area under the receiver-operating characteristic curve: MAGE (0.690, 95% CI 0.605–0.775, p < 0.001); ABG (0.581, 95% CI 0.492–0.670, p = 0.076)

The area under the ROC curve for MAGE (0.690, 95% CI 0.605–0.775, p < 0.001) was superior to that for ABG (0.581, 95% CI 0.492–0.670, p = 0.076) (Fig. 2). MAGE, but not ABG, displayed significant value in predicting short-term MACE in patients.

## Discussion

Our study shows that an elevated MAGE level during hospital is associated with a significantly higher risk of short-term MACE and cardiovascular mortality after PCI in non-diabetes patients with STEMI. Measurement of in-hospital GV by CGMS in non-diabetes patients may improve risk assessment in patients presenting with acute STEMI.

Although acute hyperglycemia on admission has clearly been associated with poor outcomes in AMI patients with or without diabetes, the prognostic value of in-hospital GV in this population has been less well established. Fluctuations of glucose seem to have more deleterious effects than sustained hyperglycemia in the development of cardiovascular complications as acute glucose excursions activate the oxidative stress [12]. More and more evidences show that glycemic variability may be an important role in resolving potential cardiovascular problems in abnormal glucose metabolism. Some researchers suggested that glucose excursions is independently related to carotid intima-media thickness and may contribute to the development of atherosclerosis in individuals with diabetes independent of other risk factors [13, 14]. In our previous study, we found that GV is an important contributing factor in the severity of coronary artery disease, which is independent of the average level of blood glucose [15]. The Verona Diabetes study reported that fasting GV is an independent predictor of mortality in type 2 diabetes patients [16]. We also found that acute glucose excursions would seem to be of greater importance than admission glucose and long-term derangements of glucose metabolism in predicting 1-year outcomes following AMI [17]. Some studies concluded that GV was a significant predictor of mortality in critically ill patients independently from mean glucose level and severity of illness [18–20]. In the present study, patients with a higher MAGE level have higher GRACE risk scores. After 3-month follow-up, a significantly higher incidence of MACE and cardiac mortality were found in those patients. Multivariate analysis disclosed that in participants, in-hospital GV (i.e. MAGE >3.26 mmol/L) was an independent predictor of MACE, but ABG was not. The results indicate that high glucose fluctuations may be associated with the risk of short-term adverse cardiovascular events in non-diabetes patients with STEMI after PCI.

Patients with admission hyperglycemia tend to experience the worst outcomes as demonstrated in several well-conducted prior studies, our study shows that increased glucose excursions should be more important. In this study, multivariate analysis shows that in-hospital GV, age, LVEF and TIMI flow after PCI were independent predictors of short-term MACE, but ABG was not. The area under the receiver-operating characteristic curve for MAGE and ABG in predicting short-term MACE shows that MAGE, but not ABG, displayed significant value in predicting short-term MACE in patients. ABG represents only a marker of point-in-time glucose status and cannot reflect the overall exposure of acute glucose swings. Glycemic disorders are not solely limited to sustained hyperglycemia but can be extended to the glycemic variability which includes both upward and downward acute glucose changes. Patients with similar mean glucose levels can have markedly different glycemic variability [21].

Although we did not address the underlying relationship between GV and cardiovascular outcomes following PCI in non-diabetes patients with STEMI, both oxidative stress and inflammation may be involved in the process. GV acutely increases oxidative stress and exaggerates inflammation [12, 22]. Some studies reported that the apoptosis of endothelial cells exposed to intermittent high glucose may be related to a reactive oxygen species (ROS) overproduction, through protein kinase C (PKC)-dependent activation of nicotinamide adenine dinucleotide phosphate-oxidase [23, 24]. In vitro studies indicate that glucose fluctuations can activate nuclear factor-κB and PKC pathway, leading to a greater expression of the adhesion molecules and excess formation of advanced glycation end-products than stable high glucose [25, 26]. These findings suggest that glucose fluctuations may augment inflammation via oxidative mechanisms closely linked to adverse outcomes. Furthermore, severe glycemic excursions may adversely affect sympathetic dysfunction which is associated with mortality and morbidity of cardiovascular disease [27]; and the thrombotic properties of platelets are increased in a hyperglycemic environment, and this can result in additional cardiovascular complications [28]. Hypoglycemia is another possible link between GV and poorer cardiovascular outcomes. Some reported that greater GV predicted more hypoglycemic episodes. Severe hypoglycemia can predict all-cause mortality in patients with diabetes [29, 30]. Hypoglycemia could induce the onset of MACE through induction of inflammation, blood coagulation abnormality, sympathoadrenal response and endothelial dysfunction [31].

Moreover, the relationship between GV and cardiovascular outcomes is still controversial. There is still an extensive debate about GV as a risk factor for MACE [32]. The trial, known as HEART2D study, failed to demonstrate that decreasing GV led to reduced MACE risk [33]. However, the study was discontinued prematurely due to too few MACE and less than expected differences in postprandial glucose values. Only one of three markers of GV was reduced with the prandial-targeted therapy and this was the newest, least established GV marker. Different aim of initial study design and measurements of GV may be important causes for explaining controversial results in these studies. Overall, more well-designed studies are warranted to investigate whether GV will play an important role in cardiovascular complications.

### Study limitations

Several study limitations should be considered in the interpretation of the results. First, diabetes was defined as known diabetes status on admission. It is well known that a number of STEMI patients have undetected diabetes mellitus, and they were not excluded in our study. Second, the sample size was relatively small, so that some subgroup comparisons may have lacked power to detect significant differences for selected variables. Third, hypoglycemia is considered as a risk factor of cardiovascular events. However, due to very low incidence of hypoglycemic episodes in our study, we didn’t include this risk factor in analysis. In addition, we examined in-hospital GV which couldn’t reflect daily GV at home. Some factors, such as different diets, operations, physical and emotional factors, which maybe affect patients’ glucose fluctuations couldn’t be analyzed. On the other hand, some limitations of CGMS should be noted including requirement for frequent calibrations, invasive techniques, complex procedure, and so on. Hence, we think that the results of the present study should be interpreted with caution. This study is hypothesis-generating and should stimulate a larger multicenter evaluation.

## Conclusions

In-hospital GV would seem to be of greater importance than ABG in predicting short-term cardiovascular outcomes in non-diabetes patients with STEMI after PCI.

## Abbreviations

MACE: major adverse cardiac events
GV: glycemic variability
AMI: acute myocardial infarction
STEMI: ST elevated myocardial infarction
ABG: admission blood glucose
MAGE: the mean amplitude of glycemic excursions
CGMS: continuous glucose monitoring system
SMBG: self-monitoring of blood glucose
GRACE: global registry of acute coronary events
CK: creatine kinase
TIMI: thrombolysis in myocardial infarction
HF: heart failure
TVR: repeat target vessel revascularization
PCI: percutaneous coronary intervention
LVEF: left ventricular ejection fraction
eGFR: estimated glomerular filtration rate
